# Broad-Snouted Caiman (*Caiman latirostris*) bite

**DOI:** 10.1590/0037-8682-0655-2021

**Published:** 2022-02-25

**Authors:** Roberto Gomes Tarlé, Vidal Haddad

**Affiliations:** 1 Pontifícia Universidade Católica do Paraná, Faculdade de Medicina, Disciplina de Dermatologia, Curitiba, PR, Brasil.; 2 Universidade Estadual Paulista, Faculdade de Medicina de Botucatu, Departamento de Infectologia, Dermatologia, Radioterapia e Diagnóstico por Imagens, Botucatu, SP, Brasil.

**FIGURE 1:**
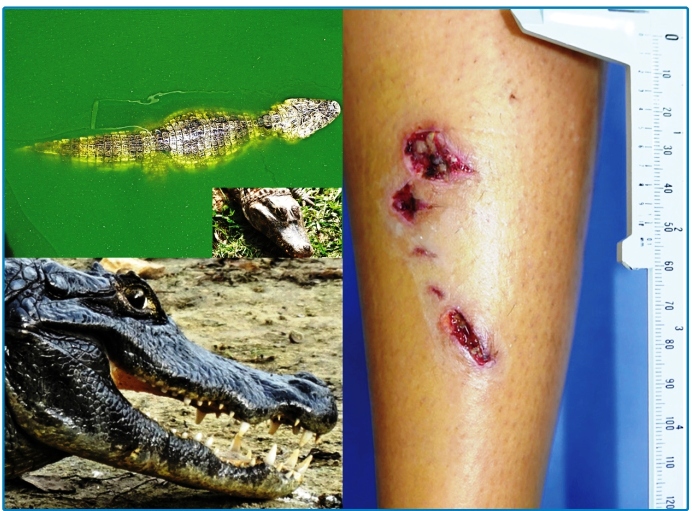
Left: Broad-Snouted Caiman (*Caiman latirostris*) and teeth of a crocodilian. Right: Multiple lacerated and perforated skin wounds on the right leg of the patient. Photos: Roberto Gomes Tarlé and Vidal Haddad Junior.

A 35-year-old female from Ilha do Mel (an estuarine complex of Paranaguá, in Southern Brazil), while walking at night on a sandy trail, was bitten by a 5 foot Broad-Snouted Caiman (Figure 1) on her right leg after a friend stepped on the alligator’s back, which was misjudged to be a tree trunk. She presented with significant bleeding and was evaluated at a local medical facility receiving antiseptic care, bandages with neomycin cream, and cephalexin 500 mg four times a day. Four days later, the patient presented with multiple perforated and lacerated skin wounds of 1 to 2 cm on her left leg (Figure 1), complaining of pain, inflammation, and purulent discharge in the wounds. The patient had no bone fractures.

*Escherichia coli* was isolated from wound cultures. The antibiotics sulfamethoxazole, trimethoprim, and ciprofloxacin were administered. She received anti-tetanus immunoglobulin and daily dressing with fusidic acid cream. Rabies prevention was not necessary because the reptiles did not transmit the disease. At 72 hours, the inflammatory signs and pain were significantly improved, and at four weeks, the lesions showed complete re-epithelialization.

The Broad-Snouted Caiman is widely distributed in Brazil, in freshwater habitats in the Southeast and South regions, and in all the coastal environments. *Caiman latirostris* is a medium-sized crocodilian, reaching up to 3.5 meters in length^1^. 

 Caiman bites must be considered polymicrobial wounds. The use of preventive antimicrobials has been recommended^2^ ^,^ ^3^. In non-hospitalized patients, the use of amoxicillin-clavulanate, sulfamethoxazole, and trimethoprim or azithromycin associated with quinolone has been advocated^2^ ^,^ ^3^.
